# Annotations

**Published:** 1893-10-21

**Authors:** 


### Annotations.

### Dr. A., Dr. B., and Dr. C.

Dr. A. treats the disease but not the patient; Dr. B. treats the symptoms, but neither the disease nor the patient; Dr. C. treats first the patient, then the disease, and last of all the symptoms." Such was the account given by a competent eye-witness of the different methods displayed by a trio of physicians gathered round the sick bed of a suffering fellow-mortal not very long ago. The patient, one would think, must come logically first. His safety, his ease, his comfort, his surroundings are naturally the things to claim earliest attention. That he have fresh air and a good bed, that he have suitable food, that he be put in the way of refreshing sleep, that the bowels, kidneys, and skin be duly considered, that the necessities of his mind be attended to; these and the like are surely the things to be put in order at the first visit, and carefully supervised and directed at every succeeding visit. Next in order comes the actual disease. What that is must be made out with certainty, or when that is impossible a temporary working hypothesis must be adopted. A physician should never leave his patient's house without having made up his mind, either as to the certainty that a patient is suffering from a particular disease, or as to the more or less decided probability; and upon that diagnosis treatment should be at any rate begun. Nothing can be so bad for a patient, and for his doctor, too, as mere haphazard treatment designed for no object, and aiming at no result. When the patient is comfortable, when the diagnosis is made, when the treatment is decided upon which is to carry the patient through his illness or some stage of it, then attention may be given to symptoms and personal worries and troubles. Our contention is, not that symptoms are unimportant, or that they are not to be treated; but that in the physician's mind at the bedside, the order of importance of the subjects under discussion is—first the patient, second the disease, third the symptoms. The man who treats the symptoms, but neither the disease nor the patient, is hardly worth calling a physician at all; the man who treats disease but disregards the patient is only half a physician; whilst the man who treats with due competency the patient, the disease, and the symptoms in this order is, in Bacon's phraseology, "a full man," and the wisest of physicians.

### The Howard Association.

Those who think that because the prisons and workhouses of to-day are infinitely better managed than were those of a century ago, therefore the work of the Howard Association is done, forget one or two elementary principles of human nature. Every person is ready to acknowledge that the "price of liberty is eternal vigilance." It is a truth of like universal import that the "price of progress is eternal effort." Such work as that of the Howard Association can never be finished so long as there are helpless orphans, vicious parents, and stupid administrators of the laws of the land. It is to be feared that helpless orphans, vicious parents, and stupid administrators of law will continue to the end of time. Two of the chief things which the Howard Association has in hand are the putting an end to the bringing up of children in workhouses, and the abolition of the punishment of imprisonment for young children. No man needs to argue the first of these propositions further than to ask himself whether or not he would like any of his own children to be brought up in a workhouse. If one might put the history of a workhouse child into a sentence it would generally run thus: "He was bred a pauper; he had an unhappy childhood; in youth he was despised; in manhood he was a failure; in old age he returned to the workhouse, and was buried in a pauper's grave." A sad lot for a brother man! The imprisonment of children of tender age often leads to consequences as terrible and as permanent as the rearing of children in workhouses—sometimes to consequences infinitely more tragical. Can anything be more foolish or more cruel than to send children, who hardly yet know right from wrong, to herd with criminals because they have committed some trifling offence against the rights of persons or property? Punish children who do wrong by all means, and punish them adequately; but inasmuch as our one object is, or ought to be, to make them good and honest, we ought not to do a single thing which will familiarise them with crime, or check their education in the virtues and decencies of life. The children of the cultured often do things which are really as vicious as many of the so-called "crimes" for which the children of the poor are sent to prison. But the public would be as horrified as the parents of such children if it were proposed to imprison them. Let us all try to put ourselves in the place of the young, the ignorant, and the badly trained, then we shall all be stanch friends of the Howard Association.

### A Whisper to Women.

There has been a marriage this week at the office of the *Times* newspaper; and the contracting parties were science and humanity. These two have been brought together by love, irresistible love: love, not so much for themselves or for one another as for certain poor helpless creatures about which they have both been for a long time deeply concerned. Science, in the person of Mr. W. H. Hudson, the distinguished naturalist, says that "Aigrettes, formed of ospreys," that is the feathers used for adorning ladies' hats and bonnets, "consist of the slender decomposing dorsal feathers of the white herons or egrets; that they are the birds' nuptial ornaments, and consequently are only to be obtained during the breeding season, when the death of the parent bird involves the death by starvation of the young in the nest." Humanity, in the person of a leader writer in the *Times*, joins hands with science in this generous protest, and pleads thus: "How long will women tolerate a fashion which involves such wholesale, wanton, and hideous cruelty as this? If their sense of humanity is too feeble, will not their native sense of maternity arouse them? . . . Let every woman who adorns herself with a dead bird, or with plumage only to be got by wanton slaughter, think first of the bright and joyous being that has died in order that she may be smart, and then of the callow fledglings lingering in desolate starvation in the ravished and deserted nest." Now ladies, the man who takes the trouble to study the manners and general progress of his own times feels a deep glow of satisfaction in witnessing this union of science and humanity in defence of helpless mother birds and their still more helpless young. But what must the same man feel when he considers that it is women, the present and future mothers of children, against whom those unhappy birds and their young have to be defended? It is women who insist upon holding the position of cruel and murderous monsters; and it is men, and such men as calm scientists and men-of-the-world newspaper writers, who plead with those cruel women, and plead in vain. Surely, here is a turning of the world upside down which would be incredible if it were not so palpably, cruelly, wickedly true. What more is there to be said? According to the science and literature of the *Times* newspaper the most cruel monsters of our own period, in our own or any other country, are the women of the better classes.

